# Incidence and Overall Survival of Malignant Ameloblastoma

**DOI:** 10.1371/journal.pone.0117789

**Published:** 2015-02-18

**Authors:** Alexandra Rizzitelli, Nicolas R. Smoll, Michael P. Chae, Warren M. Rozen, David J. Hunter-Smith

**Affiliations:** 1 Department of Plastic and Reconstructive Surgery, Frankston Hospital, Peninsula Health, 2 Hastings Road, Frankston, Victoria 3199, Australia; 2 Department of Surgery, Monash University, Level 5, E Block, Monash Medical Centre, 246 Clayton Road, Clayton, Victoria 3168, Australia; 3 Monash University Plastic and Reconstructive Surgery Unit (Peninsula Clinical School), Peninsula Health, 2 Hastings Road, Frankston, Victoria 3199, Australia; 4 Department of Surgery, School of Medicine and Dentistry, James Cook University Clinical School, Level 1, Townsville Hospital, 100 Angus Smith Drive, Douglas, Townsville Queensland 4814, Australia; National Health Research Institutes, TAIWAN

## Abstract

**Background:**

Malignant ameloblastoma, comprising metastasizing ameloblastoma and ameloblastic carcinoma, represents 1.6–2.2% of all odontogenic tumors. Due to its rare nature, malignant ameloblastoma has only been reported in the literature in small case series or case reports. Using the Surveillance, Epidemiology and End-Results (SEER) database, we have performed a population-based study to determine the incidence rate and the absolute survival of malignant ameloblastoma.

**Method:**

Using the International Classification of Diseases for Oncology (ICD-O) codes 9310/3 and 9270/3, data from the SEER database were used to calculate the incidence rate and absolute survival rate of population with malignant ameloblastoma.

**Results:**

The overall incidence rate of malignant ameloblastoma was 1.79 per 10 million person/year. The incidence rate was higher in males than females and also higher in black versus white population. The median overall survival was 17.6 years from the time of diagnosis and increasing age was associated with a statistically significant poorer survival.

**Conclusions:**

To our best knowledge, we report the largest population-based series of malignant ameloblastoma. The incidence rate was 1.79 per 10 million person/year and the overall survival was 17.6 years.

## Introduction

Benign ameloblastoma is the second most common odontogenic tumor that is histologically benign, but locally aggressive [1]. In contrast, malignant ameloblastoma is rare and constitutes 1.6–2.2% of all odontogenic tumors [2–4]. After decades of controversy [5,6], the World Health Organization (WHO) classified malignant ameloblastoma into two types: metastasizing ameloblastoma and ameloblastic carcinoma[7]. Metastasizing ameloblastoma histologically resembles the benign ameloblastoma but demonstrates metastatic spread to distant sites. Ameloblastic carcinoma exhibits malignant histological features and can be further divided into two subtypes: primary and secondary. Primary ameloblastic carcinoma arises *de novo*, while secondary ameloblastic carcinomas are a result of malignant transformation of a previously diagnosed benign ameloblastoma. In this article, the term ‘malignant ameloblastoma’ will refer to metastazing ameloblastomas and ameloblastic carcinoma together. Malignant ameloblastomas have only been reported in small case series or case reports and no population-based studies of its incidence have been reported. Metastasizing ameloblastoma is often considered to account for 2% of benign ameloblastoma[8], but the actual incidence rate is likely to be less[9]. The exact incidence rate of ameloblastic carcinoma is unknown and only approximately 100 cases have been reported in the literature[10].

The Surveillance, Epidemiology, and End Results (SEER) Program of the National Cancer Institute is a population-based cancer database covering approximately 28% of the United States (US) population and collects epidemiological information of patients with cancer.

In this study, we have extracted the incidence rate and absolute survival from 293 malignant ameloblastoma cases reported in the SEER database. To our knowledge, this is the largest series of malignant ameloblastomas analyzed and the first report of incidence rate.

## Methods

### The Surveillance, Epidemiology and End Results (SEER) Database

A case listing session was opened in the SEER database. Our search parameters included the SEER 18 Regs Research Data + hurricane Katrina Impacted Louisiana Cases, Nov 2013 sub (1973–2011 varying) and the International Classification of Diseases for Oncology (ICD-O) codes 9310/3 (metastasizing ameloblastomas) and 9270/3(ameloblastic carcinomas). For analysis, we utilized the age groups recommended from the Adolescent and Young Adult Oncology (AYAO) Progress Review Group: children (0–15 years old), young adults (16–39), adults (40–64), and elderly (≥65)[11]. The treatments recorded are only those which were offered to the patient at diagnosis.

### Incidence Rate

Incidence rates and confidence intervals (CI) were obtained from the SEER 9 registries. Incidence rates were age-standardized to the US standard population in 2000, which is the latest standard population released by SEER for use with the SEER*stat software. Incidence rate ratios were used to measure the effect size of variables on the incidence rate. Unsmoothed age-standardized rates were presented in tables and smoothed estimates were plotted on graphs. Moving averages with a 4, 1, and 4 window were used in order to reduce random fluctuation. This provided a clearer view of the underlying behavior of the data. The incidence rate ratio (IRR) represents the incidence rate per unit of population (10 millions) of the age or race category divided by the incidence rate of the base.

### Overall Survival

Survival data were obtained from the SEER 18 registries. Survival was defined as time from diagnosis until death due to all causes (overall survival). Kaplan–Meier estimates of survival were calculated and the logrank test used to determine survival differences between the age groups. Cox proportional hazards regression was used to calculate hazard ratios.

### Statistical Analysis

All statistical analyses were performed with Stata software (Version 13; StataCorp, College Station, TX). To describe the patient cohort, simple proportions were used along with Pearson’s chi-squared test to identify significant imbalances. Multivariable modeling was performed using forced entry methods as these methods are likely superior to stepwise and other sequential entry/exit methods [12]. Poisson regression was used to analyse the statistical significance of age-adjusted incidence rate differences per gender. Type I error was 0.05 for all tests. P-values less than 0.05 were deemed significant.

## Results

### Study Cohort

The extraction from the SEER 18 registries yielded 293 patients with a median age of 52 years (interquartile range 31). The characteristics of the patients included in this analysis are summarized in Table 1. There were significantly more males than females diagnosed with malignant ameloblastoma (62% of the cohort) and white ethnicity constituted 66% of the study group. There was an imbalance in the site of disease affected depending on the patient’s age: in 75% of children and young adults, the malignant lesion was found in the “bones of skull”, which most likely indicates the maxilla. In contrast, the mandible was the most commonly affected area in the elderly (41%). Regarding treatment, surgical excision was the most common mode of therapy (89%) followed by radiotherapy (15%).

**Table 1:** Baseline characteristics of patients.

	**Children and young adults**		**Adults**		**Elderly**		**Total**		**P-value**
	No	%	No	%	No	%	No	%	
**Total**	89	30	115	40	89	30	293	100	-
**Sex**									
**Male**	46	52	81	70	54	61	181	62	0.023
**Female**	43	48	34	30	35	39	112	38	
**Year of diagnosis**									
**1973–1984**	7	8	14	12	11	12	32	11	0.176
**1985–1994**	19	21	15	13	13	15	47	16	
**1995–2004**	37	42	38	33	40	45	115	39	
**2005–2013**	26	29	48	42	25	28	99	34	
**Site**									
**Bones of skull**	67	75	73	63	42	47	182	62	0.003
**Mandible**	19	22	31	27	36	41	86	29	
**Other sites**	3	3	11	10	11	12	25	9	
**Race**									
**White**	45	51	83	72	66	74	194	66	0.004
**Black**	28	31	20	17	18	20	66	23	
**Other or unknown**	16	18	12	10	5	6	33	11	
**Radiation**									
**None, refused or unknown**	82	92	98	85	69	78	249	85	0.024
**Radiation**	7	8	17	15	20	22	44	15	
**Treatment**									
**No surgery**	3	3	14	12	16	18	33	11	0.008
**Surgery**	86	97	101	88	73	82	260	89	

P-values obtained with Pearson chi-squared test.

### Incidence

**Fig 1:**
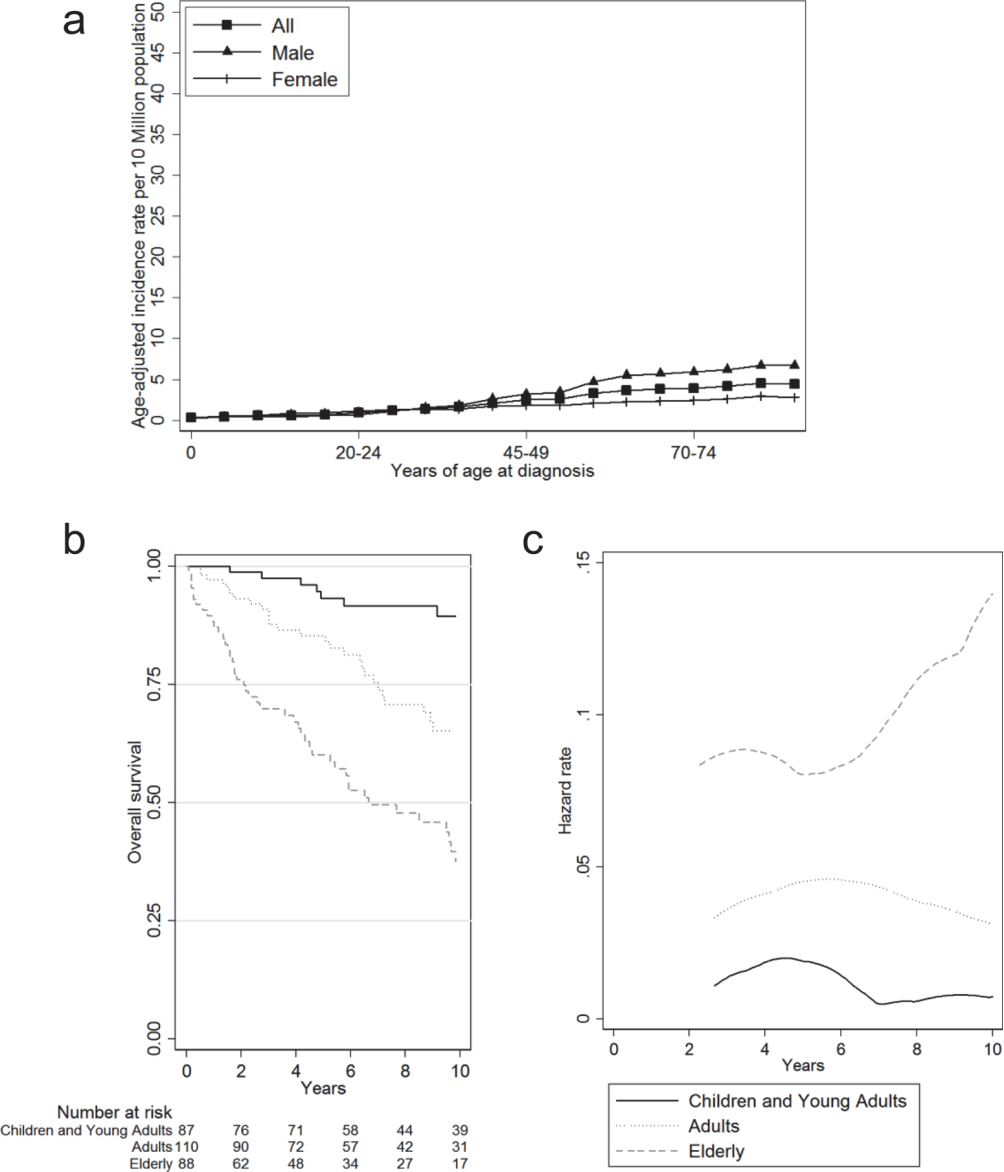
Incidence rate and overall survival. (a) Smoothed incidence rates (per 10 million) across patient ages at diagnosis, by sex. (b) Kaplan-Meier curves and (c) smoothed hazard rates by age group.

The overall incidence rate of malignant ameloblastoma was 1.79 per 10 million person/year (Table 2). Both incidence rates and rate ratios increased with age. Children and young adults were affected at a rate of 0.84 per 10 million person/year; adults, 2.5 per 10 million person/year and elderly, 4.4 per 10 million person/year. In the male population, the incidence rate was 2.27 per 10 million person/year and in female, 1.42. As a result, the incidence rate ratio was 1.59 males affected for every female. The incidence rate was also significantly higher in the black population than in the white. Fig. 1A showed how the difference in the incidence rates for males and females was dependent on the age at diagnosis, with the sex differences becoming apparent as age increased, rather than being homogenous throughout the ages. Nevertheless, this apparent difference was not statistically significant.

**Table 2:** Incidence rates and incidence rate ratios (IRR) per 10 million population.

	**Counts (%)**	**Incidence rate (95% CI)**	**IRR (95% CI)**
**Total**	164 (100)	1.79 (1.53–2.09)	0.72 (0.54–0.96)
**Children and young adults**	48 (29)	0.84 (0.61–1.11)*	0.33 (0.22–0.49)
**Adults**	69 (42)	2.5 (1.95–0.32)	Base
**Elderly**	47 (29)	4.4 (3.23–5.85)*	1.75 (1.18–2.58)
**Female**	69 (42)	1.42 (1.1–1.8)	Base
**Male**	95 (58)	2.27 (1.83–2.78)*	1.59 (1.15–2.21)
**White**	108 (66)	1.46 (1.19–1.76)	Base
**Black**	41 (25)	4.71 (3.32–6.47)*	3.2 (2.16–4.72)
**Other**	11 (7)	1.14 (0.57–2.08)	0.78 (0.37–1.48)
**Unknown**	4 (2)	N/A	N/A

Data obtained from SEER 9 registries.

* p<0.05.

N/A, not-applicable.

### Survival

Overall, the median survival was 17.6 years and was not significantly affected by sex or race (Table 3). Children and young adults fared the best with 89% survival at 10 years and a relatively flat survival curve (Fig. 1B). In contrast, the elderly had the poorest percentage of survivors at 10 years (60%) and the shortest median survival (6.6 years). Moreover, the elderly population was 9.6 (95% CI 5–18.1) times as likely to die as the young adult population after adjusting for multiple confounders (but not accounting for background mortality). This resulted in the steepest survival curve of all age groups (Fig. 1B).

**Table 3:** Overall survival.

		% Survivors at _ years				Median survival	HR (95% CI)	P-value
	No	0.5	1	5	10	(years)		
**All**	285	97	95	80	64	17.6	N/A	N/A
**Age group**								
**Children & young adults**	87	100	100	93	89	N/A	Base	N/A
**Adults**	110	98	97	85	65	22	2.9 (1.5–5.6)	0.002
**Elderly**	88	92	87	76	60	6.6	9.6 (5–18.1)	<0.001
**Sex**								
**Female**	109	97	96	78	66	18.6	Base	N/A
**Male**	176	96	94	81	62	15	1.2 (0.8–1.8)	0.33
**Race**								
**Black**	63	100	98	79	70	19.5	Base	N/A
**White**	191	95	93	78	59	14.9	1.4 (0.8–2.3)	0.21
**Other or unknown**	31	100	96	92	86	23	0.6 (0.2–1.7)	0.35
**Radiation**								
**None, refused, unknown**	241	97	96	84	69	18.9	Base	N/A
**Radiation**	44	93	86	55	31	5.7	2.7 (1.7–4.2)	<0.001
**Treatment**								
**No surgery**	30	93	82	54	29	5.4	Base	N/A
**Underwent surgery**	255	97	96	83	67	18.6	0.3 (0.2–0.6)	<0.001
**Site**								
**Mandible**	176	97	96	85	67	19.5	Base	N/A
**Bones of skull**	84	97	95	75	62	13.6	1.7 (1.1–2.6)	0.016
**Other sites**	25	92	88	62	44	9.2	2.3 (1.3–4.3)	0.007

Univariate Cox proportional hazards ratio model. N/A: not applicable.

Using a univariate analysis, we showed that patients with malignant lesion to the “bones of skull” or “other sites’ were respectively 1.7 and 2.3 times as likely to die as patients with mandibular lesions (Table 3). Interestingly, this did not show statistical significance using a multivariate approach (Table 4). It was difficult to assess the effect of a treatment (radiation or surgery) on all-cause mortality despite being statistically significant because of the inherent selection bias and lack of protocols dictating the administration of such therapies. The variables were included in the final model as part of the forced-entry method.

**Table 4:** Overall survival.

	**HR (95% CI)**	**P-value**
**All**	N/A	N/A
**Age group**		
**Children & young adults**	Base	N/A
**Adults**	2.42 (1.22–4.76)	0.011
**Elderly**	7.71 (3.98–14.95)	<0.001
**Sex**		
**Female**	Base	N/A
**Male**	1.01 (0.66–1.55)	0.08
**Race**		
**Black**	Base	N/A
**White**	1.17 (0.7–1.97)	0.54
**Other or unknown**	0.84 (0.31–2.31)	0.75
**Radiation**		
**None, refused, unknown**	Base	N/A
**Radiation**	2.26 (1.42–3.6)	0.001
**Treatment**		
**No surgery**	Base	N/A
**Underwent surgery**	0.45 (0.25–0.8)	0.006
**Site**		
**Mandible**	Base	N/A
**Bones of skull**	1.2 (0.77–1.85)	0.42
**Other sites**	1.68	0.1

Multivariate Cox proportional hazards ratio model. N/A: not applicable.

An important negative finding on univariable and multivariable analysis was that sex and race did not have survival differences: HR 1.01 (95%CI 0.66–1.55) for males compared to females and HR 1.17 (95%CI 0.7–1.97) for whites compared to black people (Table 4).

Fig. 1C demonstrated a divergent hazard rate curves after 6 years of follow-up, indicative of a fork-type covariate-by-follow-up interaction.

## Discussion

Perusing the SEER data, our study demonstrated that the overall incidence rate of malignant ameloblastoma was 1.79 per 10 million persons per year with male and black population predominance. The incidence rate increased as individual got older and elderly patients who received a diagnosis of malignant ameloblastoma were nearly 10-times more likely to die than children and young adults. Part of this increased HR in elderly could be explained by the increasing background mortality or competing risks of death in this population. With what is currently known about aging, it is reasonable to assume that a certain proportion of the effect of age on mortality is due to confounding factors, such as age-associated diseases or decreased physiological reserve found in the elderly population. More specifically, competing risks of death are likely to have caused an over-estimation of the cumulative incidence of death and an underestimation of overall survival related to this tumor [13]. Answering this question with appropriate precision requires a larger sample size analyzed using the methods of relative survival as used elsewhere [14–18].

The majority of malignant ameloblastomas occurred in males rather than females and this results confirmed previous studies showing the male to female ratio to be between 2.3 and 5[10,19,20]. The cause for this increased incidence of malignant ameloblastomas in males compared with females has not been investigated or reported, and remains unknown. The incidence rate of developing a malignant ameloblastoma was higher in the black population. To the best of our knowledge, this has never been reported before and merits further epidemiological studies. Such race imbalance was suggested in 1978 by Shear and Singh, but their study only concerned cases of benign ameloblastoma[21]. It remains to be explored if this difference is determined by genetic or environment factors.

Our univariate (but not multivariate) analysis showed there was a significant increase in HR for patient diagnosed with a malignant ameloblastoma affecting the “bones of skull” and “other sites”. Ameloblastoma most commonly affects the mandible and maxilla, rarely other sites[22]. The SEER database only recorded the site of ameloblastomas as either the mandible or the “bones of skull”. Hence, the latter was assumed to comprise mostly maxillary tumors. This results was surprising as the literature reports the mandible as the preferred site of disease development [10,20,23]. However, most case reports published to date are from India, Japan and China. It is thus possible that this anatomical distribution imbalance is a unique feature of the U.S. population.

There is controversy regarding radiotherapy as a treatment option for ameloblastoma as it is generally considered a radio-resistant tumor. One study reviewed ten patients with ameloblastomas treated with megavoltage irradiation and concluded that ameloblastoma was not an inherently radio-resistant tumor and that properly applied megavoltage irradiation had a useful role in the management[24]. However, there has not been any well-documented evidences concerning the true radio-responsiveness of these tumors since that study, and some authors have doubt on its effectiveness, unless the tumor is inoperable[25].

One of the limitations of our study is that the SEER data represents only a portion of the U.S. population and may generate sampling bias. Previous studies demonstrated that people living within the registries captured by the SEER are more affluent, have lower unemployment rate, and are more urbanized[26]. Moreover, the benign ameloblastoma is known to be more prevalent in Africa and Asia, and relatively infrequent amongst Caucasians [27–29]. Whether this is true for malignant ameloblastomas remains to be demonstrated. Another limitation of utilizing the SEER data for rare tumor studies is that there was no central pathology review to validate the diagnosis. However, the SEER Program is still considered one of the most comprehensive nationwide databases and the data is frequently utilized by investigators worldwide for peer-reviewed publications[30].

For the purpose of our study, we did not separate malignant ameloblastoma into its two subtypes (metastasizing ameloblastoma and ameloblastic carcinoma). Individually, metastasizing ameloblastoma (n = 184) and ameloblastic carcinoma (n = 109) did not record a sufficient number to allow sound statistical analysis and survival modeling.

Finally, we demonstrated a divergent hazard rate curves after 6 years of follow-up, indicative of a fork-type covariate-by-follow-up interaction (formal testing not done due to sample size limitations) [16]. This was an indication that hazards were no longer proportional after 6 years of follow-up and thus our interpretation of the Cox-proportional hazards model (hazard ratios) must be limited to 6 years. This could partly be due to disease characteristics, physiologic reserve or most likely the increased expected mortality as the elderly person ages.

## Conclusion

To our best knowledge, we report here the largest series of malignant ameloblastomas in the literature and demonstrate its overall incidence rate as 1.79 per 10 million persons per year with male and black population predominance.
